# Initial radiological signs of dentofacial deformity in juvenile idiopathic arthritis

**DOI:** 10.1038/s41598-021-92575-4

**Published:** 2021-06-23

**Authors:** Peter Stoustrup, Michelle Sys Traberg, Louise Hauge Matzen, Mia Glerup, Annelise Küseler, Troels Herlin, Thomas Klit Pedersen

**Affiliations:** 1 Section of Orthodontics, Institute for Oral Health, Aarhus University, Vennelyst Boulevard, 9-11, Aarhus, Denmark; 2 Section of Radiology, Aarhus University, Aarhus, Denmark; 3 Department of Pediatrics, Aarhus University Hospital, Aarhus, Denmark; 4 Department of Oral and Maxillofacial Surgery, Aarhus University Hospital, Aarhus, Denmark

**Keywords:** Medical research, Rheumatology

## Abstract

Juvenile idiopathic arthritis (JIA) is the most common rheumatic disease in childhood and the temporomandibular joint (TMJ) is often involved. TMJ arthritis in growing individuals can cause deformation of facial skeleton (dentofacial deformity) and TMJ components (TMJ deformity). Treatment outcome hinges on early initiation of anti-inflammatory treatment and orthopaedic treatment with dental splints. The aim of the present study was to characterize the radiological signs of dentofacial deformity in patients with a JIA-induced need for orthopaedic treatment. We retrospectively studied 96 patients with JIA and 20 non-JIA controls to identify the initial radiological signs of JIA-induced dentofacial deformity leading to initiation of orthopaedic treatment. We found that initial radiological signs of dentofacial deformities were subtle and characterized by minor mandibular asymmetry and occlusal plane steepening. Radiological findings of TMJ deformity associated with initial dentofacial deformity were frequent and characterized by condylar articular surface flattening (OR 8.42), condylar subcortical cyst (OR 5.94), condylar surface erosion (OR 5.38) and condylar deviation in form (OR 25.39). Radiological signs of TMJ deformity were also documented in TMJs considered “healthy” during initial clinical and radiological examination. This study presents new knowledge of importance for early diagnosis of dentofacial deformity in JIA. Early diagnosis of dentofacial deformity is important as treatment outcome is greatly influenced by timely initiation.

## Introduction

Juvenile idiopathic arthritis (JIA) is the most common rheumatic disease in children and adolescents with an incidence rate of 12.8–15/100,000 children per year in the North European countries^1,2^. Arthritis in the temporomandibular joint (TMJ) is a common finding in JIA with a reported prevalence of approximately 40 per cent^3,4^. TMJ arthritis can lead to deformation of joint components, which is accompanied by orofacial signs and symptoms having a negative impact on quality of life^5–9^. These orofacial manifestations of JIA can present from disease onset and persist into adulthood^6,9^. Progression of JIA manifestations can also occur in systemically well-treated patients where TMJ inflammation has resolved^10^. Recent consensus-based recommendations have therefore highlighted the importance of focusing on two aspects during orofacial monitoring of patients with JIA: the actual TMJ inflammation (defined “TMJ arthritis”) as well as the clinical and radiological complications following current/previous TMJ inflammation (defined “TMJ involvement”)^11,12^.

In skeletally immature subjects with JIA, TMJ involvement may lead to deformity of the TMJ component and facial skeleton (defined “TMJ deformity” and “dentofacial deformity”)^13,14^. Extrapolated evidence suggests that TMJ involvement and dentofacial deformity are rather frequent JIA complications. A recent regional-based cohort study found that 35 per cent of skeletally immature subjects with JIA developed radiological signs of dentofacial deformity within 5 years after onset of JIA disease^9^. In addition, recent population-based data found that 61 per cent of subjects with JIA had radiological signs of TMJ deformities 17 years after disease onset^6^.

Management of dentofacial deformities consists of orthopaedic appliances (dental splints) in combination with fixed orthodontic appliances in growing subjects and surgical correction in full-grown individuals; systemic treatment with synthetic and biological disease-modifying antirheumatic drugs (DMARDs) aims to minimize progression of dentofacial deformity^15–19^. The outcome of orthopaedic treatment is greatly influenced by timely initiation^19^. However, little is known about the morphological changes characterizing the progression from “healthy” mandibular appearance into the state of arthritis-induced dentofacial deformity. Hence, improved knowledge about initial radiological signs of dentofacial deformity and the associated deformation of the intra-articular structures of the TMJ (TMJ deformity) is important to aid timely diagnosis and prompt initiation of management. Recently, interdisciplinary consensus-based recommendations have recommended the use of cone-beam computed tomography (CBCT) for assessment of TMJ and dentofacial deformity^11^. Based on 3D CBCTs, the objectives of the present study were: (1) to identify the initial radiological signs of dentofacial deformity leading to initiation of orthopaedic treatment in patients with JIA and TMJ involvement. (2) To characterize the initial intra-articular radiological signs of TMJ deformity associated with initial signs of facial deformity in patients needing orthopaedic treatment.

## Materials and methods

The present cross-sectional study was based on data from the Aarhus University Craniofacial Clinic at the Section of Orthodontics, Aarhus University, Denmark. The project and the collection and storing of data was approved by the licensing committee of the Danish Health Authorities (3-3013-2283/1) and the Danish Data Protection Agency (1-16-02-745-17) in accordance with Danish national research regulations. Consent was waived by the licensing committee of the Danish Health Authorities (3-3013-2283/1) to retrospectively collect and use the data from the existing electronic patient files of the eligible subjects. Data collection and handling of data was conducted in accordance with relevant guidelines and national regulations.

The present study adheres to the standardised terminology of orofacial conditions in JIA, which defines: “TMJ arthritis” as active inflammation in the TMJ; “TMJ involvement” as clinical and/or radiological abnormalities, presumed to be the result of TMJ arthritis; “Dentofacial deformity” as abnormality in growth, development, structure and/or alignment of the facial bones and dentition; and “TMJ deformity” as abnormality in growth, development or structure of the osseous and/or soft-tissue components of the TMJ^11^.

### Participants

Eligible participants were identified retrospectively from the medical records of the Aarhus University Regional Craniofacial Clinic. The vast majority of patients diagnosed with JIA are longitudinally followed at the Regional Craniofacial Clinic for tax-funded examination and management of JIA-related orofacial conditions. The JIA population followed at the clinic is representative for the overall JIA population of the western part of Denmark. Prior to data collection, study groups were subdivided according to the diagnosis of TMJ involvement and dentofacial deformity, which was initially based on an overall assessment of findings derived from the orofacial examinations and CBCT examination preceding the decision to initiate standard orthopaedic treatment with a dental splint. The study groups were defined as follows: (1) a JIA group with bilateral TMJ involvement and initial signs of dentofacial deformity leading to initiation of orthopaedic treatment (JIA+Bilat). (2) A JIA group with unilateral TMJ involvement and initial signs of dentofacial deformity leading to initiation of orthopaedic treatment (JIA+Uni). Together, the two JIA groups with dentofacial deformity and TMJ involvement (JIA+Bilat and JIA+Uni) were classified “JIA+”. (3) A JIA group with no clinical signs of TMJ involvement and normal dentofacial morphology without a need for orthopaedic treatment (JIA−). IV) A non-JIA control group seeking regular orthodontic care at the Section of Orthodontics, Aarhus University, Denmark.

JIA+ classified patients seen at the Aarhus University Craniofacial Clinic from March 2005 to April 2017 were included based on the following three criteria: (1) JIA diagnosis according to the ILAR criteria (20); (2) clinical and/or radiological findings documenting TMJ involvement and incipient dentofacial deformity/asymmetry to such an extent that initiation of orthopaedic treatment with a dental distraction splint was initiated; and (3) a high-quality full-face CBCT taken for diagnostic purposes and treatment planning before initiation of the orthopaedic splint treatment. Patients in the JIA+Uni group were eligible for inclusion only if they showed no clinical and/or radiological signs of progression into bilateral TMJ involvement at the first CBCT follow-up scan > 2 years after the first CBCT examination.

An age-matched group of JIA patients without clinical and radiological signs of dentofacial deformity (JIA-) was included based on the following criteria: (1) a diagnosis of JIA according to the ILAR criteria (20). (2) A high-quality full-face CBCT taken for diagnostic purposes in the period from 2005 to 2017 of comparable age with the two JIA+ groups. (3) No need for orthopaedic treatment based on clinical and CBCT findings; e.g. normal facial morphology (no deformity), normal TMJ function, and normal radiological TMJ findings taking into account the morphological variations that TMJ can exhibit in the background population. Group III patients (JIA−) were eligible for inclusion only if they did not develop clinical signs of TMJ involvement within the first 2 years after the CBCT examination used in the present study.

In addition, a control group was also included. The control group consisted of non-JIA patients referred to the Section of Orthodontics for general orthodontic treatment (e.g. missing teeth, ankylosis and tooth impaction). The inclusion criteria for the control group were patients with: (1) no previous history of temporomandibular disorders, facial traumas, syndromes or congenital anomalies involving the facial skeleton, or the presence of rheumatic conditions. (2) A high-quality full-face CBCT performed as a part of the pre-orthodontic examination and treatment planning.

### Patient characteristics

Demographic data were obtained by reviewing medical records. These data included: age at the time of CBCT examination, sex, JIA subcategory according to the ILAR criteria^20^, disease duration, medication at the time of CBCT examination, and presence of orofacial symptoms at the time of CBCT examination (Table 1).

**Table 1:** Patient characteristics at time of radiological examination (n = 116).

Cohort characteristics	Controls (n = 20)	JIA− (n = 22)	JIA+Uni (n = 42)	JIA+Bilat (n = 32)
**Sex**				
Female	12	12	32	25
Male	8	10	10	7
Mean age at CBCT examination, years (SD)	10.6 (0.9)^a^	9.5 (2.4)	8.6 (2.7)	9.2 (2.5)
**JIA subcategories, number (%)**				
Oligoarticular	0	5 (22)	21 (50)	14 (44)
Polyarticular (RF neg)	0	9 (41)	12 (29)	15 (47)
Systemic	0	2 (9)	1 (2)	0
Psoriatic	0	1 (5)	1 (2)	1 (3)
Enthesitis-related arthritis	0	1 (5)	0	0
Unknown	0	4 (18)	7 (17)	2 (6)
TMJ symptoms at time of CBCT examination (%)	0	0	24 (57)	17 (53)
**Prescribed medication at time of CBCT examination (%)**				
No medication	20 (100)	7 (32)	18 (43)	12 (38)
NSAID mono	0	5 (23)	8 (19)	8 (25)
MTX monotherapy	0	3 (14)	12 (28)	5 (16)
Biologics monotherapy	0	1 (5)	2 (5)	2 (6)
Biologics+MTX	0	5 (23)	2 (5)	3 (9)
Biologics+systemic corticosteroids	0	1 (5)	0	1 (3)
Other	0	0	0	1 (3)
**JIA disease duration at time of CBCT (%) (years)**				
< 3	–	22 (100)	36 (86)	22 (69)
3–5	–	0	4 (10)	7 (22)
> 5	–	0	2 (5)	3 (10)

^a^Controls were significantly older than the JIA groups.

*JIA* Juvenile idiopathic arthritis, *CBCT* cone-beam computed tomography, *RF* rheumatoid factor, *TMJ* temporomandibular joint, NSA*I*DS NON-steroidal anti-inflammatory drugs, *MTX* methotrexate.

### Assessment of dentofacial deformity—primary outcome

A NewTom 3G or 5G (CEFLA, Italy) 18 × 16 cm field of view was used for the CBCT examinations. The image acquisition parameters included a scanning time of approximately 18 s, active radiation for 3.6 s with settings of 110 kV and 3–7 mA (radiation dose was 190 microSv). All CBCT scans were constructed with a 0.3 mm isotropic voxel dimension. The 3D evaluation was performed using the following programs: Mimics (Mimics 18.0; Materialize) and OnDemand (Cybermed).

Dentofacial morphology was assessed according to a validated protocol by Stoustrup et al. for assessment of dentofacial deformity from JIA^21^. This original protocol consists of 21 outcome variables. In the present study, 13/21 of the original outcome variables were included for the morphometric assessment of facial morphology; the 13 items represent variables originally rated to be of “moderate to high importance” for dentofacial assessment in JIA^21^. The outcome variables are specifically described in Table 2 and are presented in online supplement S2–S4. Six of the included variables describe facial asymmetry by comparing inter-side differences in linear distances and angles (variables 1–6). The “asymmetry” side of each patient was defined as the side with the smallest total posterior height (variable 1). The side with the largest height was subtracted from the smaller side, resulting in a negative value as a description of inter-side asymmetry in millimetres. The “asymmetry” side, defined by outcome variable 1, was further used to calculate the degree of asymmetry in variables 2–6^21^. The following outcome variables used reference planes to describe mandibular asymmetry (variable 7), mandibular inclination (variables 8, 10), mandibular sagittal position, (variables 9, 12), anterior–posterior face height (variables 11) and mandibular chin-point deviation (variables 13). The supplemental material holds information about landmarks and constructed planes used in the morphometric analysis and illustrates all 13 outcome variables.

**Table 2:** Summary of morphometric measures used in the radiological assessment of dentofacial deformity.

Reference number (no.)	Morphometric measures	Growth deviation assessed
	**Inter-side diff. in bilateral linear distances**	
1	Total posterior mandibular height	Difference in mandibular vertical development
2	Condylar height	Difference in condyle height
3	Mandibular basal length	Difference in distance from gnathion to gonion
4	Maxillary occlusal canting	Canting of the maxillary occlusal plane measured at molars
5	Mandibular occlusal canting	Canting of the mandibular occlusal plane measured at molars
	**Inter-side difference in bilateral angles**	
6	Gonion angle	Difference in gonion angle
	**Angles between predefined planes**	
7	Mandibular axial angle (z-axis asymmetry)	Canting of the mandibular lower border measured at gonion
8	Mandibular inclination	Assessment of mandibular inclination and rotation
9	Mandibular sagittal position	Sagittal position of the mandible
10	Mandibular occlusal inclination	Inclination of mandibular occlusal plane
11	Anterior/posterior lower face height ratio	Anterior lower facial development
	**Miscellaneous**	
12	Wits appraisal	Difference between distance of A-point and B-point to coronal molar construction plane
13	Transverse distance, gnathion, to midsagittal plane	Distance from gnathion to sagittal plane

Definitions of landmarks and planes used for the assessments are included in the supplemental material.

Prior to assessment of all included patients, intra-rater (MST) reproducibility was established based on duplicate assessments of all 13 outcome variables in 30 random patients from the four study groups.

### Assessment of TMJ deformity—second outcome

Assessment of TMJ deformity was conducted at the joint level based on the radiological material used for assessment of dentofacial deformity in the first part of the study. In the second part of the study, each TMJ was assessed and scored individually and assigned to one of two groups: TMJs diagnosed with involvement during the initial clinical and radiological examination were classified “TMJ_pos_”; TMJs without a clinical diagnosis of TMJ involvement were classified “TMJ_neg_”. Hence, in the statistical analysis, patients in the JIA+ uni group were represented with two different units, TMJ_pos_ and TMJ_neg_. Regardless of joint status, the TMJ morphology of all included joints was assessed by LHM in a random fashion (block randomization). The radiological TMJ evaluation was conducted according to the protocol by Ahmad et al.^22^; this protocol consists of 14 unique items describing various morphological deviations. Eleven items relate to condylar radiological appearance and the relationship between condyle and fossa/tuberculum. The remaining three items relate to deviations of the temporal fossa/eminence^22^. Each TMJ was rated according to all 14 items based on a dichotomous outcome (presence of feature: yes/no). The assessor was blinded to group assignment during evaluation. Prior to assessment of all eligible TMJs, intra-rater reproducibility was established based on duplicate assessment of 20 random TMJs representing all four study groups. Scans with artefacts or limited resolution of the relevant TMJ structures were excluded for this second part of the study.

### Statistics

#### Reproducibility

An intra-class correlation coefficient (ICC, one-way random effect model) was used to assess intra-rater reproducibility of the 13 morphometric measures of dentofacial deformity based on duplicate assessment in 30 random patients^23^. An ICC of ≥ 0.70 was considered acceptable. Cohen’s kappa coefficient (κ) was used to assess intra-rater reproducibility of the 14 items in the TMJ deformity scoring system by Ahmad et al., based on duplicate assessment in 20 random CBCTs.

#### Assessment of dentofacial deformity

A Shapiro–Wilk test was performed to test for normal distribution of the 13 outcome variables included in the morphometric analysis of dentofacial deformity. Outcome variables presenting normal distribution were evaluated using ANOVA test with unpaired Student’s t tests serving as post-ANOVA tests. Only outcome variables indicating a significant inter-group difference in the primary ANOVA test were further analysed with the unpaired Student’s t test. Outcome variables that were not normally distributed were analysed using a Kruskal–Wallis test. Only outcome variables with an indication of a significant inter-group difference in the primary Kruskal–Wallis test were further analysed with a Wilcoxon signed-rank test. The level of significance was set at p = 0.05.

#### Assessment of TMJ deformity

Descriptive statistics was used to establish the prevalence of findings related to the 14-item scoring system within the TMJ_pos_ and TMJ_neg_ groups.

#### Associations between TMJ morphological findings and TMJ involvement

The dichotomous outcomes were expressed as odds ratios in a multivariate logistic regression model to identify traits of TMJ deformity associated with initial signs of JIA-induced dentofacial deformity.

### Other

A Kruskall–Wallis test was used to assess inter-group differences in JIA disease duration at the time of CBCT examination.

## Results

We included a total of 116 participants for morphometric analysis of whom 74 were assigned to the JIA+ group. Based on baseline clinical and radiological findings of TMJ involvement and dentofacial deformity, these participants were further subcategorized into the JIA+Uni group (n = 42, 57%) or the JIA+Bilat group (n = 32, 43%); 22 age-matched patients with JIA but without apparent TMJ involvement were assigned to the JIA- group. Twenty non-JIA participants were identified for the control group.

### Patient characteristics

Patient characteristics are displayed in Table 1. No significant inter-group difference in sex distribution was found. At the time of CBCT examination, the control group had a significantly higher mean age (10.6 years, SD 0.9) than the three JIA groups: JIA− mean 9.5 years (SD 2.4 years), JIA+Uni 8.6 years (SD 2.7 years) and JIA+Bilat mean 9.2 years (SD 2.5 years). Oligoarticular and RF-neg polyarticular JIA were the most common subcategories. Together they represented 63% of the JIA− group, 79% of the JIA+Uni group and 91% of the JIA+Bilat group. The majority (55%) of the JIA+ patients reported the presence of orofacial symptoms at the time of CBCT examination. Seventeen of the JIA patients received biological treatment at the time of CBCT examination (17.7%): five JIA patients received biological monotherapy, ten patients received biological treatment in combination with methotrexate, and two patients received biological treatments in combination with systemic corticosteroids.

### Assessment of dentofacial deformity

**Figure 1:**
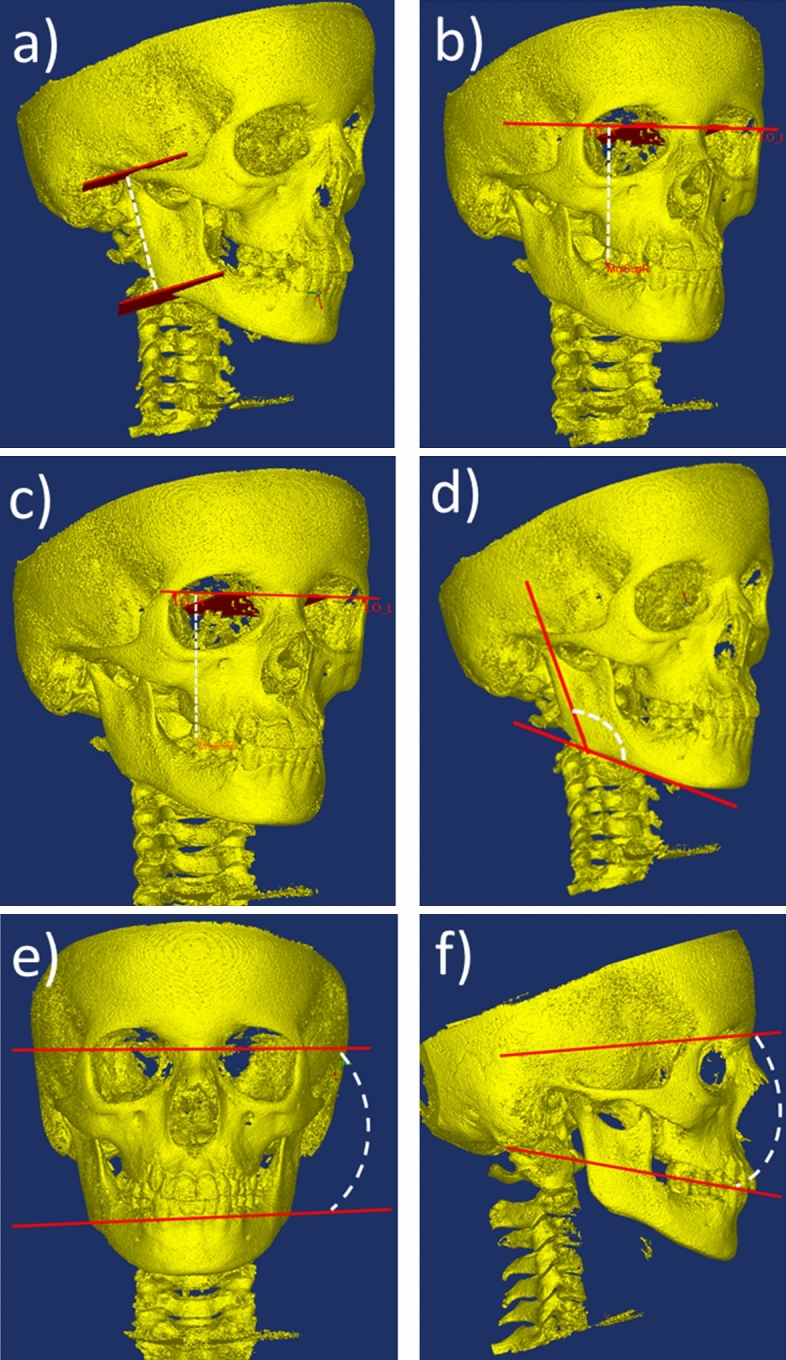
Assessment of dentofacial deformity. Figures only display morphometric measures with a significant inter-group difference (Table 3). Supplement material display all 13 outcome measures. (**a**) Total posterior height (inter-side difference). (**b**) Maxillary occlusal canting (inter-side difference in distance from axial plane to maxillary 1st molar). (**c**) Mandibular occlusal canting (inter-side difference from axial plane to mandibular 1st molar). (**d**) Gonion angle (inter-side difference). (**e**) Mandibular axial angle (angle between the two reference planes). (**f**) Mandibular occlusal inclination (angle between the two reference planes).

The intra-rater reproducibility was considered acceptable: the ICC values ranged from 0.70 to 0.98 in 12/13 of the morphometric outcomes. Only the ICC of the mandibular basal length was considered poor (0.08). Ten of the 13 morphometric measures were normally distributed. Table 3 displays the 10 normally distributed outcome variables and the results of the statistical inter-group comparison. Three outcome variables were not normally distributed: total posterior mandibular height (Table 3, ref. No 1), maxillary occlusal canting (Table 3, ref. No 4) and mandibular occlusal canting (Table 3, ref. No 5). Figure 1 displays the six outcome variables with a statistical inter-group difference in the primary ANOVA tests (all 13 outcome variables are presented in the online supplemental material S3). The JIA+Uni group was more asymmetric than the other three groups as seen from significantly larger inter-side differences in total posterior mandibular height (Table 3, ref. No 1; Fig. 1a), maxillary occlusal canting (Table 3, ref. No 4; Fig. 1b) 4), gonion angle (Table 3, ref. No 6; Fig. 1d) and mandibular axial angle (Table 3 ref. No 7; Fig. 1e). A minor degree of dentofacial asymmetry was observed in the TMJ+Bilat group as seen from a significant inter-side difference in total posterior mandibular height (Table 3 ref. No1; Fig. 1a) and maxillary occlusal canting (Table 3 ref. No 4; Fig. 1b) compared with controls and the JIA− group. In addition, both the JIA+Uni and the JIA+Bilat had a greater mandibular occlusal inclination (Table 3, ref. No 10; Fig. 1f) than controls and the JIA- group. In general, comparable dentofacial morphology was observed between the control group and the JIA− group.

**Table 3:** Morphometric assessment of dentofacial deformity. Negative values indicate degree of inter-side asymmetry in millimeters.

Ref. no.	Morphometric measures	C Control Mean, (*SD; range*)	JIA1 JIA− Mean, (*SD; range* )	JIA2 JIA+Uni Mean, (*SD; range* )	JIA3 JIA+Bilat Mean (*SD; range* )	Primary test (ANOVA/Kruskal Wallis tests)	Post-tests^a^ (T test/Mann Whitney)
	**Inter-side diff. in bilateral linear distances**						
1	Total posterior mandibular height^b^	− 1.5, *(1.4; − 5.0: − 0.1)*	− 1.2, *(1.1; − 4.3: − 0.1)*	− 3.9, *(2.9; − 15.9:− 0.67)*	− 2.8, *(2.7; − 12.7:− 0.1)*	0.0001	C = JIA1 > JIA3 > JIA2
2	Condylar height	0.9, *(4.6; − 9.0: 9.9)*	− 0.9, (5.5; *− 14.5: 9.8)*	− 1.9, *(6.9; − 18.4: 9.8)*	− 2.0, *(8.8; − 17:6: 15.8)*	0.41	–
3	Mandibular basal length	1.1, *(2.2; − 1.7: 5.8)*	0.5*, (1.7; − 2.3: 4.0)*	− 0.2, *(2.8; − 6.5: 6.4)*	0.9, (3.6; *− 8.7: 6.5)*	0.18	–
4	Maxillary occlusal canting^b^	− 0.4, *(1.7; − 5.8: 2.0*)	0.2, *(1.3; − 2.8: 2.3)*	− 1.5, *(1.6; − 7.7: 0.7)*	− 0.7, *(1.4; − 4.7: 1.9)*	0.0001	JIA1 > JIA3 > JIA2, (C = JIA1 and JIA3)
5	Mandibular occlusal canting^b^	− 0.01, *(0.9; − 1.8: 1.1)*	0.2, *(1.4; − 3.2: 2.5)*	− 1.5, *(1.6; − 7.3: 0.5)*	− 0.5, *(1.5; − 4.3: 2.6)*	0.0001	JIA1 > JIA3 > JIA2, (C = JIA1 and JIA3)
	**Inter-side difference in bilateral angles**						
6	Gonion angle	0.01, *(1.9; − 2.9: 3.7)*	0.6, *(2.4; − 3.2: 6.2)*	− 2.1, *(2.9; − 9.3: 4.6)*	0.2, *(4.1; − 9.1: 8.9)*	0.0014	C = JIA1 = JIA3 > JIA2
	**Angles between predefined planes**						
7	Mandibular axial angle (z-axis asymmetry)	1.2, *(0.9; 0.04: 3.1)*	1.0, *(0.6; 0.01:2.4)*	2.1, *(1.7; 0.1:6.2)*	1.5, *(1.3; 0.01: 5.6)*	0.018	C = JIA1 < JIA2, (JIA2 = JIA3)
8	Mandibular inclination	28.6, *(4.8; 20.5: 37.7)*	29.0, *(4.8; 18.6: 37.4)*	30.5, *(5.8; 19.3: 44.7)*	32.8, *(7.6; 17.9: 53.4)*	0.053	–
9	Mandibular sagittal position	80.4, *(4.3; 72.8: 86:8)*	79.8, *(3.6; 74.0: 85.9)*	79.3, *(3.6; 70.3: 87.4)*	78.3, *(5.7; 65.1: 89.9)*	0.37	–
10	Mandibular occlusal inclination	12.5, *(5.8; 0.8: 22.4)*	13.9, *(4.7; 7.9: 27.4)*	16.1, *(4.7; 4.9: 27.7)*	17.4, *(6.3; 5.9: 29.8)*	0.007	JIA1 < JIA3, C < (JIA2 = JIA3)
	**Anterior/posterior face height ratios**						
11	Posterior/anterior lower face height ratio	0.7, *(0.04; 0.7: 0.8)*	0.7, * (0.4; 0.7: 0.8)*	0.7, *(0.1; 0.6: 0.8)*	0.7, *(0.5; 0.6: 0.8)*	0.55	–
	**Miscellaneous**						
12	Wits appraisal	0.9, *(4.6; − 8.5: 8.7)*	1.5, *(3.2; − 3.8: 7.4)*	0.8, *(2.5; − 4.7: 7.2)*	1.9, *(3.2; − 5.1: 9.4)*	0.46	–
13	Transverse distance, gnathion to midsagittal plane	− 0.4, *(1.7; − 4.0: 3.4)*	0.6, *(2.2; − 3.8: 5.5)*	− 0.6, *(2.8; − 9.0: 5.6)*	− 0.4, *(2.6; − 7.6: 4.8)*	0.48	–

^a^Post-tests were conducted only in variables with a significant inter-group difference in the primary test. ^b^Not normally distributed.

### Assessment of TMJ deformity from TMJ involvement

The intra-rater kappa values all exceeded 0.85, indicating acceptable agreement. Not all 14 morphological items included in the scoring system were represented in the duplicate assessment of TMJ morphology. Therefore, it was not possible to calculate kappa values for outcome variables with reference numbers 1, 2, 8, 9, 11 and 13 (Table 4).

**Table 4:** Assessment of temporomandibular joint (TMJ) deformity. A multivariate logistic regression model is used to express intergroup differences.

Outcome variables with reference numbers	No of TMJs	TMJs with involvement (TMJ_pos_) n = 94	TMJs without involvement (TMJ_neg_) n = 116	OR (95% CI)	p
1. Condylar hypoplasi	210	0	0		
2. Condylar hyperplasia	210	0	0		
3. Condylar articular surface flattening	210	75 (80%)	37 (32%)	8.42 (4.07–17.43)	< 0.0001
4. Condylar subcortical sclerosis	210	2 (2%)	1 (1%)		
5. Condylar subcortical cyst	210	23 (25%)	6 (5%)	5.94 (2.21–15.95)	< 0.0001
6. Condylar surface erosion	210	38 (40%)	13 (11%)	5.38 (2.53–11.44)	< 0.0001
7. Condylar osteophyt	210	7 (8%)	8 (7%)	1.09 (0.38–3,12)	0.88
8. Condylar generalized sclerosis	210	0	0		
9. Loose joint body	210	0	0		
10. Condylar deviation in form	210	17 (18%)	1 (1%)	25.39 (2.97–216.89)	< 0.0001
11. Bony ankylosis	210	0	0		
12. Fossa articular surface flattening	210	3 (3%)	0		
13. Fossa subcotical sclerosis	210	1 (1%)	0		
14. Fossa surface erosion	210	6 (6%)	0		

Level of significance was 0.05.

*OR* odds-ratio, *CI* confidence interval.

**Figure 2:**
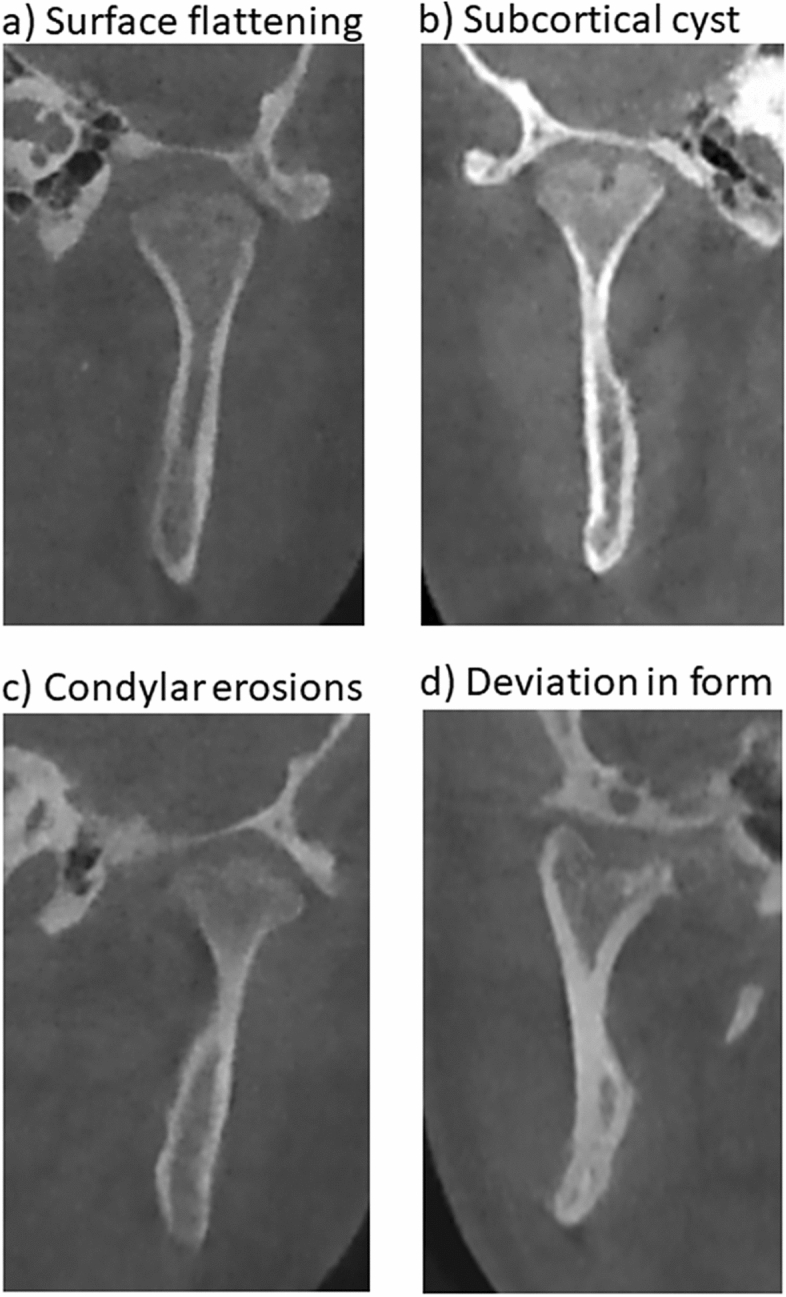
Assessment of temporomandibular joint (TMJ) deformity: figures only morphometric outcome measures with a significant inter-group difference (Table 4). (**a**) Condylar surface flattening, definition: a loss of the rounded contour of the surface^22^. (**b**) Condylar subcortical cyst, definition: a cavity below the articular surface that deviates from normal marrow pattern^22^. (**c**) Condylar surface erosion, definition: loss of continuity of articular cortex^22^. (**d**) Deviation in form, definition: condylar deviation in form is defined as a departure from normal shape, such as concavity in the outline of the cortical plate, and not attributable to flattening, erosive changes, osteophytes, hyper or hypoplasia^22^.

From the morphometric analysis of dentofacial deformity in the first part of this study, 116 individuals (232 TMJs) were eligible for assessment of TMJ deformity. However, 11 patients (22 TMJs) were excluded from this second part of the study due to CBCT artefacts and inadequate radiological quality. The excluded radiological material represented 10 TMJ_pos_ and 12 TMJ_neg_. After exclusion, a total of 210 TMJs were included in the assessment of TMJ deformity: 94 TMJ_pos_ and 116 TMJ_neg_. Table 4 presents the prevalence of TMJ deformity within the two groups together with the results of the multivariate logistic regression analysis. Articular surface flattening of the mandibular condyle was the most predominant radiological finding in the TMJ_pos_ group; observed in 80% compared with 32% in the TMJ_neg_ group. Condylar surface erosions were the second most prevalent finding in the TMJ_pos_ group; observed in 40%. The following radiological signs of TMJ deformity were significantly associated with initial signs of dentofacial deformity from JIA involvement of the TMJ condyle: articular surface flattening (OR 8.42), subcortical cyst (OR 5.94), surface erosion (OR 5.38) and condylar deviation in form (OR 25.39) (Table 4). Figure 2 displays these four radiological findings. Importantly, TMJs previously deemed not to be involved and not to have caused dentofacial deformity (TMJ_neg)_ during the clinical/radiological evaluation also, although less frequently, presented radiological signs of TMJ deformity. When assessed by a trained maxillofacial radiologist in a blinded, standardized fashion, articular surface flattening (32%), subcortical cysts (5%) and surface erosions (11%) were also seen in the TMJ_neg_ group. Deformities related to the fossa of the temporal bone were rarely observed and were represented only in the TMJ_pos_. Osteophytes were registered with the same frequency in the TMJ_pos_ (8%) and TMJ_neg_ (7%) group.

## Discussion

Within the past decade, increased effort has been devoted to establishing clinical, radiological and imaging standards to aid early diagnosis of TMJ arthritis/involvement and ameliorate its associated dentofacial consequences^10,12,24–26^. The rationale for this effort is, among others, to avoid development of dentofacial deformities induced by TMJ involvement. Research has indicated a non-linear relationship between the severity of JIA-induced facial deformity and the severity of the intra-articular TMJ deformity^14^. This means that patients with pronounced TMJ deformity can present with only minor dentofacial deformity. Conversely, severe dentofacial asymmetry can also be seen in patients with minor radiological signs of TMJ deformity^27^. This situation underlines the importance of studying initial radiological signs of dentofacial deformity together with signs of TMJ deformity since neither can stand alone in the radiologic study of JIA patients with TMJ involvement. This is the first CBCT-based study to investigate the initial radiological signs of dentofacial deformity and the associated signs of TMJ deformity caused by JIA.

### Dentofacial deformity

The characteristics of dentofacial deformities caused by long-term TMJ arthritis range from minor asymmetries to micrognathia^15,21,28^. In the present study, dentofacial deformity refers to pathology patients receiving orthopaedic treatment for a deviating dentofacial growth pattern. According to our standard procedure, tax-funded orthopaedic treatment is applied as soon as clinical and radiological findings demonstrate incipient dentofacial deformity beyond normal variation^19^. In the present study, subtle radiological signs of mandibular asymmetry constituted the most prevalent finding of early dentofacial deformity in JIA patients with both unilateral and bilateral TMJ involvement. A potential explanation for the presence of dentofacial asymmetry in patients with bilateral TMJ involvement is a difference in the onset of involvement between the two TMJs.

According to Liukkonen et al., mild dentofacial asymmetry is a natural phenomenon in the general population; still, a normal range has not been established^29^. The pronounced variation in dentofacial asymmetry heightens the risk of misinterpreting naturally occurring dentofacial asymmetry with early signs of JIA-induced dentofacial deformity. However, since dentofacial deformities vary depending on the underlying aetiologies, it is important to understand the characteristics of deformities related to TMJ involvement in JIA. In the present study, early signs of dentofacial asymmetries from TMJ involvement were characterized by unilateral shortening of the mandibular ramus with a cant of the mandibular basis and the occlusal plane upwards to the side with the shortest vertical development. This is a characteristic asymmetry observed in JIA (Fig. 1abe) and in other non-JIA asymmetries originating from an inter-side difference in the posterior part of the mandible^30^. These characteristic findings should therefore be the focus in clinical and radiological examination of patients with JIA. Early dentofacial deformity was not characterized by distinct mandibular retrognathia/micrognathia, which is considered a hallmark for long-term bilateral TMJ involvement^31^. For this reason, clinical assessment of facial morphology (symmetry and profile) is recommended as an important aspect of routine full-body health assessment of subjects with JIA^12,32^. Supporting the value of the clinical monitoring of dentofacial deformity, we have previously shown a good correlation between hard-tissue and soft-tissue dentofacial asymmetry^33^. Facial asymmetry in JIA is most pronounced in the lower part of the face where deviations exceeding 2 mm can be accurately identified at the clinical soft-tissue facial examination^33,34^.

### TMJ deformity

The radiological signs of TMJ deformities associated with the initial signs of arthritis-induced facial deformity range from mild variation in shape (e.g. condylar flattening) to severe deformity (e.g. erosions and morphological deviations). According to Peck et al., early stages of TMJ arthritis may not present radiological signs of TMJ deformity^35^. However, Peck et al. described that, if present, TMJ involvement caused by “systemic arthritides” is characterized by radiological signs of subchondral cysts, erosions, generalized sclerosis and osteophytes^35^. The initial radiological signs of TMJ deformity identified in the present study are somewhat in agreement with the findings by Peck et al. in terms of subcortical cysts and condylar erosions. However, we found no evidence to support that generalized sclerosis and osteophytes characterize early radiological signs of TMJ deformity associated with dentofacial deformity in JIA. Condylar osteophytes occurred equally in both groups, indicating that this finding is not specifically associated with TMJ involvement in JIA.

According to Mao et al., mechanical functional loading of a healthy TMJ can impact the intra-articular condylar growth cartilage, resulting in morphological deviations such as flattening^36^. Hence, condylar flattening alone is not a sensitive radiological indication of JIA TMJ involvement since this was seen in 32% of subjects in the TMJ_neg_ group. In addition, mechanical overloading can also cause minor cortical degeneration (e.g.). This may explain why 11% of the TMJs with no clinical diagnosis of TMJ involvement (TMJ_neg_) had signs of condylar erosions^37^. Overall, our findings document a noteworthy overlap of radiological signs of TMJ deformity between “healthy TMJs” and arthritis-induced “TMJ involvement”. Beside mechanical loading, radiological signs of TMJ deformity could also potentially emerge from the fact that subchondral cortical bone is under development in children and adolescents, which may hamper precise radiological evaluation of TMJ deformity^38^.

Our findings corroborate with the recent progressive scoring system for osseous TMJ deformity from JIA proposed by Kellenberger et al.^24^. In this MRI-based scoring system, “mild”/”moderate” radiological signs of TMJ deformity are characterized by flattening, whereas more severe deformities (grade 3 and 4) are characterized by erosions and deviations in condylar form^24^. In agreement with this, 80% of the TMJ_pos_ in the present study had articular surface flattening equivalent to a “mild”/“moderate” deformity score in the Kellenberger grading system. Radiological signs of severe deformity such as condylar surface erosions and condylar form deviations (equivalent to a score 3 and 4 in the Kellenberger scoring system) were less prevalent in the TMJ_pos_ group.

In growing individuals condylar erosions may not be “erosions” in the pathological sense (destruction of cartilaginous and bony surface) but rather demineralization of the cartilage. We have previously suggested that the different radiological findings may represent different stages of the TMJ arthritis process, where flattening may represent a “stable” phase, whereas radiological signs of erosions and deviation in form may represent a reaction to an active inflammatory phase^14^. Importantly, TMJ arthritis per se does not necessarily destroy the joint (which “erosions” imply) but probably contributes to altered growth and development of the mandibular condyle. The pathological development of dentofacial deformity is nowadays considered a complex multifactorial condition involving condylar growth retardation, TMJ deformity, degeneration, TMJ dysfunction and mechanical overloading and dentofacial compensation^27^.

### Clinical applicability

The present study demonstrates the general challenges related to the diagnosis of TMJ involvement in the daily clinical settings. Assignment to JIA+Uni and JIA+Bilat was based on standardized clinical and radiographic evaluation aiming to outline the individual need for orthopaedic and/or medical treatment at the time examination. The JIA− group had received identical standardized clinical and radiological protocols without any indication of TMJ involvement or dentofacial deformity. Future studies need to further elucidate the processes related to progression of “healthy” joints with a minor degree of morphological variation (e.g. flattening) into a state of TMJ pathology. The present study supports the importance of a multidimensional, standardized orofacial examination approach where diagnostic and treatment decisions are based on patients’ symptoms, clinical findings, radiological findings of TMJ arthritis and TMJ deformity, and findings of dentofacial deformity^12^.

### Limitations

The fact that minor dentofacial asymmetry is a naturally occurring phenomenon hampers assessment of early radiological signs of dentofacial deformity. In the present study, minor asymmetries were also seen in controls and JIA patients without TMJ involvement. Previously, Arvidsson et al. defined craniofacial growth disturbances as deformities exceeding two standard deviations from the mean findings of non-JIA controls^31^. To avoid excluding relevant radiological signs of dentofacial deformities at an early stage, we did not include this definition in the present study. Instead, we decided to make a quantitative assessment to capture the subtle inter-group differences in dentofacial deformities from early TMJ involvement. Non-surgical orthopaedic splint treatment was initiated in all patients following the diagnosis of dentofacial deformity, indicating an initial, clinically conspicuous deviation of dentofacial morphology in patients requiring orthopaedic treatment. Another limitation is the cross-sectional nature of the present study, which does not allow us to assess whether TMJ_neg_ joints with minor TMJ deformity would produce dentofacial deformity; nor does it allow us to assess if condylar remodelling (“healing”) would occur over time in the TMJ_pos_ group, as previously described^39,40^.

The lack of documentation of TMJ arthritis by contrast-enhanced MRI also represents a limitation. However, in aspects of dentofacial deformity, this limitation may be of minor importance. The development of TMJ and dentofacial deformity should be seen as a product of TMJ inflammation, condylar growth disturbance and mechanical loading^41,42^. Within the past two decades, much research has been devoted to exploring the association between TMJ inflammation and the development of dentofacial deformity. Rather less attention has been paid to the contribution of mechanical overloading and suboptimal TMJ function caused by JIA-induced TMJ deformity. Recent findings by Bollhalder et al. elucidate the impact of TMJ deformity on condylar growth. Bollhalder et al. found that the presence of baseline TMJ deformity impacts mandibular development to a greater extent than the presence of baseline TMJ inflammation in medically treated patients^16^. A recent explanatory model has proposed how mechanical overloading may also lead to a progression of dentofacial deformity in otherwise well-treated patients with no signs of current TMJ inflammation^27^.

In the scoring of TMJ deformity we have used the term “subchondral cyst” in accordance with Ahmad et el.^22^. However, fluid and soft-tissue cannot be detected by radiographic image analysis. Hence, in the present study, a subchondral cyst may not be a “true cyst” as seen in MRI analysis. We acknowledge this limitation.

Important strengths of the present study are the use of CBCT-based material, standardized definitions and standardized 3D morphological evaluation of dentofacial deformity and the large patient material.

In conclusion, initial radiological signs of arthritis-induced dentofacial deformities are subtle and characterized by dentofacial asymmetry due to unilateral shortening of the mandibular ramus with a cant of the mandibular basis and the occlusal plane upwards to the side with the shortest vertical development. In addition, initial signs of dentofacial deformity from bilateral TMJ involvement are characterized by occlusal plane steepening. Radiological signs of TMJ deformity associated with incipient dentofacial deformity in JIA are characterized by the presence of condylar articular flattening, subcortical cysts, condylar surface erosions and deviation in condylar form. However, these radiological findings may also be present, though to a lesser extent, in patients without JIA-related TMJ involvement. A multidimensional, standardized examination approach is recommended where treatment decisions are based on clinical findings of TMJ dysfunction, imaging/radiological findings of TMJ arthritis and TMJ deformity, and findings of dentofacial deformity.

## Supplementary Information

Supplementary Information 1.

## Data Availability

All relevant data is presented in the article. By Danish regulations, radiological data is considered third-party patient owned data. All data is available from the corresponding author upon preceding approval from the Danish Patient Safety authorities.
