# Supplementary material for: Initial radiological signs of dentofacial deformity in juvenile idiopathic arthritis
Source: Sci Rep. 2021 Jun 23;11:13142. doi: 10.1038/s41598-021-92575-4 (PMC8222246; doi:10.1038/s41598-021-92575-4)
Supplement: Supplementary file 1 — Supplementary Information 1. [file 41598_2021_92575_MOESM1_ESM.docx]

Supplementary information:

Initial radiological signs of dentofacial deformity in juvenile idiopathic arthritis

Peter Stoustrup^1^*, Michelle Sys Traberg^1^, Louise Hauge Matzen^2^, Mia Glerup^3^, Annelise Küseler^1,4^, Troels Herlin^3^, Thomas Klit Pedersen^1,4^.

1. Section of Orthodontics, Aarhus University, Denmark
2. Section of Radiology, Aarhus University, Denmark
3. Department of Pediatrics, Aarhus University Hospital, Aarhus, Denmark
4. Department of Oral and Maxillofacial Surgery, Aarhus University Hospital, Aarhus, Denmark

*Corresponding author: Peter Stoustrup. Mail: [pstoustrup@dent.au.dk](mailto:pstoustrup@dent.au.dk), phone: 0045 61 33 44 64. Address: Vennelyst Boulevard, 9-11, Section of Orthodontics, Institute for Oral health, Aarhus University

| **S1:** Definition of landmarks and planes in the morphometric analysis. | | |
| --- | --- | --- |
| **Anatomical landmark** | **Definition** | **Abbreviation** |
| Sella | The centre of the hypophyseal fossa | **S** |
| Nasion | Midpoint between maxillary-nasal-frontal right and left junction chin | N |
| Basion | The most anterior-inferior point on the margin of the foramen magnum | Ba |
| Pogonion | The most anterior point of the mandible in relation to the coronal plane | Pg |
| A point | Deepest concavity on the anterior part of the maxilla at sagittal plane level in relation to the coronal plane | A |
| Gnathion | The lowest point on the lower border of the chin | Gn |
| B point | Deepest concavity on the anterior part of the mandible at sagittal plane level in relation to the coronal plane | B |
| Anterior nasal spine | The most anterior point of the anterior maxillary spine | ANS |
| Condylion, R | Most superior point at the midpoint of the condyle right | CoR |
| Condylion, L | Most superior point at the midpoint of the condyle left | CoL |
| Incisura, R | Lowest point in the concavity between processus coronoideus and processus condylaris- right | IncR |
| Incisura, L | Lowest point in the concavity between processus coronoideus and processus condylaris- left | IncL |
| Gonion, R | Midpoint on the posterior-inferior ramus - Midpoint on the sagittal and coronal curvature right | GoR |
| Gonion, L | Midpoint on the posterior-inferior ramus - Midpoint on the sagittal and coronal curvature left | GoL |
| Latero-Orbital point, R | Zygomatico-frontal suture at the mesial aspect of the orbital wall, right | LO_R |
| Latero-Orbital point, L | Zygomatico-frontal suture at the medial aspect of the orbital wall left | LO_L |
| Midpoint upper incisors | Midpoint (incisal edge) between superior incisors | InS |
| Midpoint lower incisors | Midpoint (incisal edge) between inferior incisors | InInf. |
| Midpoint between incisors | Vertical midpoint between upper and lower incisors | InS-InInf. |
| Cusp upper molar, R | Disto-Facial cusp 1 upper molar, right | MolSupR |
| Cusp upper molar, L | Disto-Facial cusp 1 upper molar, left | MolSupL |
| Cusp lower molar, R | Disto-Facial 1 lower molar, right | MolInfR |
| Cusp lower molar, L | Disto-Facial cusp 1 lower molar, left | MolInfL |
| **Plane** | **Definition** | **Abbreviation** |
| 1. | S-N- perpendicular to Axial plane | Sagittal S-N-BA |
| 2. | S-Lo_R-Lo_L | Axial plane |
| 3. | S-perpendicular to the Sagittal S-N-BA - perpendicular to the Axial plane | Coronal plane |
| 4. | Gor-Gol-Gn | Mandibular axial plane |
| 5. | MolSupR- MolSupL – InS-InInf. | Combined occlusal plane |
| 6. | MolSupR-MolSupL-InS | Maxillary occlusal plane |
| 7. | MolInfR- MolInfL-InInf | Mandibular occlusal plane |
| 8. | MolSupR perpendicular to Combined occlusal plane and Sagittal S-N-BA | Molar coronal Construction plane |
| 9. | Gor-GoL-perpendicular to the Coronal plane | Gonion Axial construction plane |
| 10. | GoR-GoL- perpendicular to the Axial plane | Gonion Coronal construction plane |
| 11. | N – Pogonion - perpendicular to Sagittal S-N-BA | Mandibular protrusion plane |
| 12. | N – A-point – perpendicular to Sagittal S-N-BA | Maxillary protrusion plane: |
| **Side-specific planes** | **Definition (one in each side)** | **Abbreviation** |
| 1. | Go-Gn-perpendicular to the Axial plane | Mandibular Construction plane |
| 2. | Co-Go-Gn | Ramus sagittal plane |
| 3. | Co-Go-perpendicular to the Ramus sagittal plane | Ramus coronal plane |
| 4. | Through Co - perpendicular to Ramus sagittal plane -perpendicular to Ramus Coronal plane | Ramus Axial plane 1 |
| 5. | Through Inc- perpendicular to Ramus sagittal plane -perpendicular to Ramus Coronal plane | Ramus Axial plane 2 |
| 6. | Through Go- perpendicular to Ramus sagittal plane -perpendicular to Ramus Coronal plane | Ramus Axial plane 3 |

| **S2.** Definition of morphometric measures. | | |
| --- | --- | --- |
| **Reference number of morphometric measure (No.)** | **Morphometric measures** | **Definition** |
|  | **Inter-side difference in bilateral linear distances** | **Inter-side difference: largest side subtracted the “asymmetry side” No 1-11)** |
| 1. | Total posterior mandibular height 1 (mandibular level) | Distance from the Ramus axial plane 1 to Ramus axial plane 3 through Co-Go |
| 2. | Condylar height | Distance between Co and Ramus axial plane 2 |
| 3. | Mandibular basal length | Go-Gn |
| 4. | Maxillary occlusal canting | Distance from Axial plane to MolSup |
| 5. | Mandibular occlusal canting | Distance from Axial plane to MolInf |
|  | **Inter-side difference in bilateral angles** |  |
| 6. | Gonion angle | Angle between Ramus Coronal plane and Mandibular Axial plane (inter-side ratio right/left) |
|  | **Angles between predefined planes** |  |
| 7. | Mandibular axial angle (z-axis asymmetry) | Angle between Gonion axial Construction Plane and Axial plane |
| 8. | Mandibular inclination | Angle between Mandibular Axial plane and Axial plane |
| 9. | Mandibular sagittal position | Angle between Axial plane and Mandibular protrusion plane |
| 10. | Mandibular occlusal plane inclination | Angle between Axial plane and Mandibular occlusal plane |
|  | **Anterior/posterior face height ratios** |  |
| 11. | Anterior/posterior lower face height ratio (Cranial level) | Distance from Axial plane to Go (ave..R + L ) divided by distance from Axial plane to Gn |
|  | **Miscellaneous** |  |
| 12. | Wits appraisal | Distance between A-point and molar coronal Construction Plane (minus) distance B-point to molar coronal Construction Plane |
| 13. | Transverse distance, gnathion, to midsagittal plane (Y-axis asymmetry) | Distance between Gnathion perpendicular to Sagittal plane |

#### **S3**. Illustrations of the 13 morphometric measures used for assessment of dentofacial deformity.

1. Total posterior mandibular height. Inter-side difference in mandibular vertical development


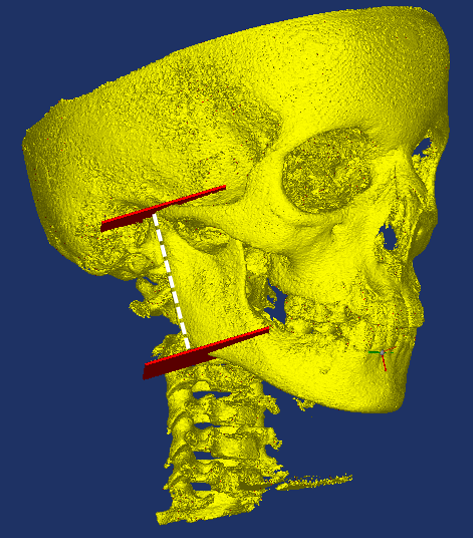


2. Condylar height. Inter-side difference in condyle height


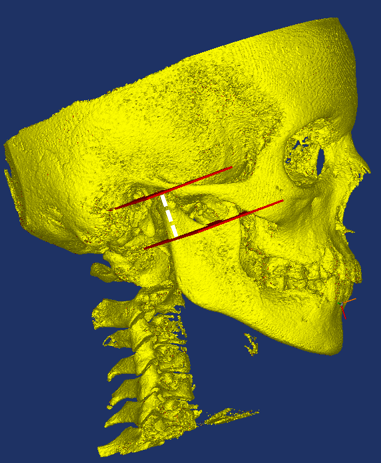


3. Mandibular basal length. Inter-side difference in distance from gnathion to gonion


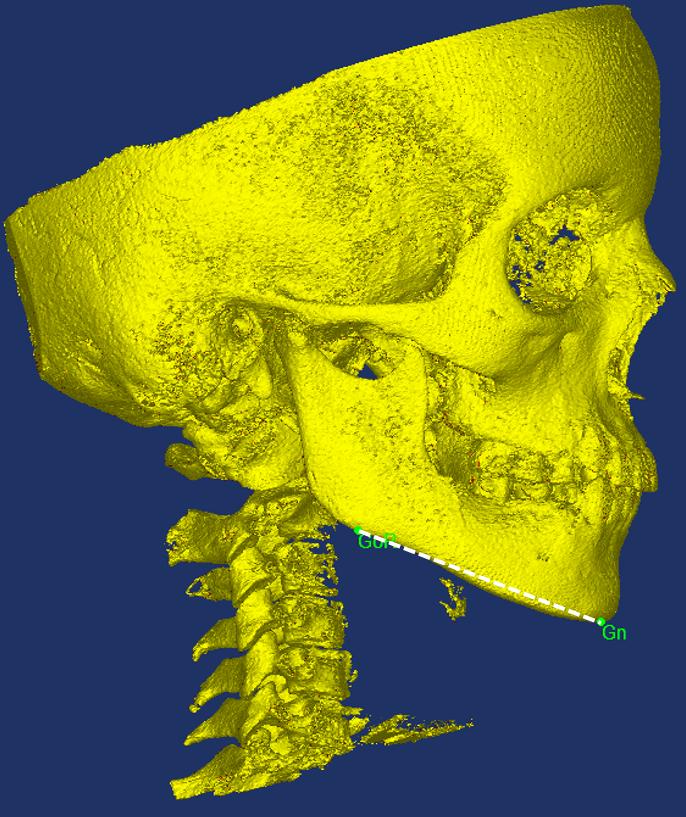


4. Maxillary occlusal canting. Canting of the maxillary occlusal plane measured at molars


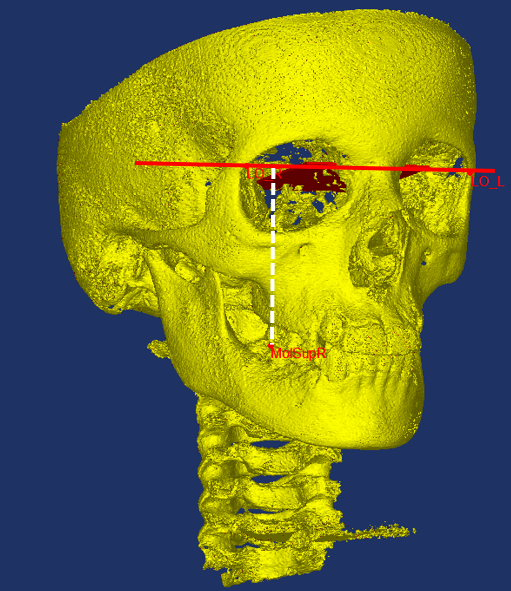


5. Mandibular occlusal canting. Canting of the mandibular occlusal plane measured at molars


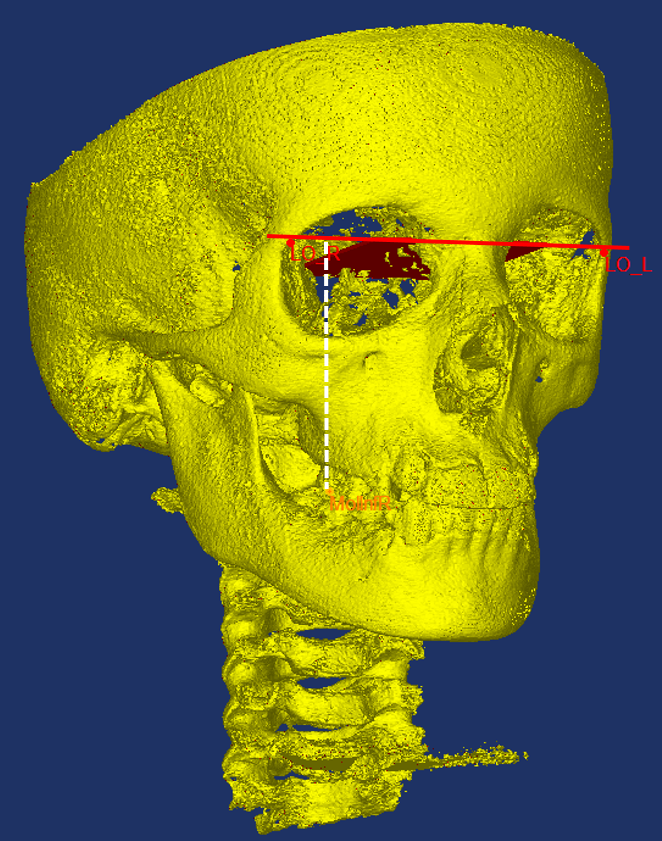


6. Gonion angle. Inter-side difference in gonion angle


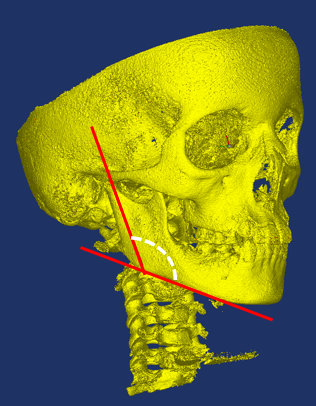


7. Mandibular axial angle (z-axis asymmetry). Canting of the mandibular lower border measured at gonion


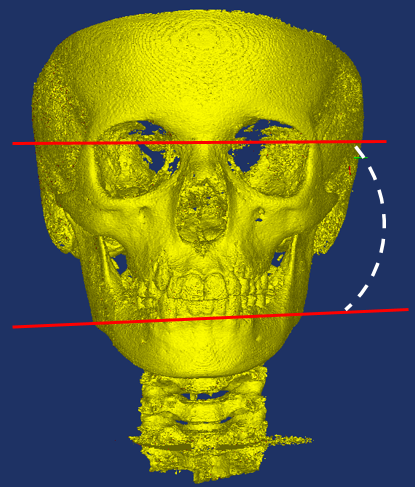


8. Mandibular inclination. Assessment of mandibular inclination and rotation


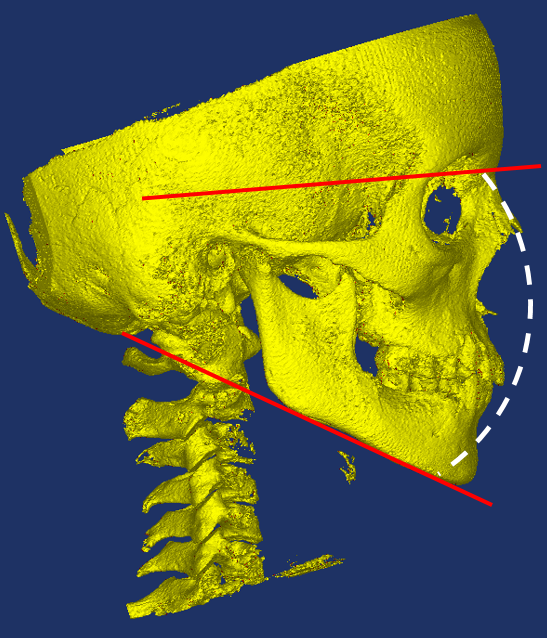


9. Mandibular sagittal position. Sagittal position of the mandible


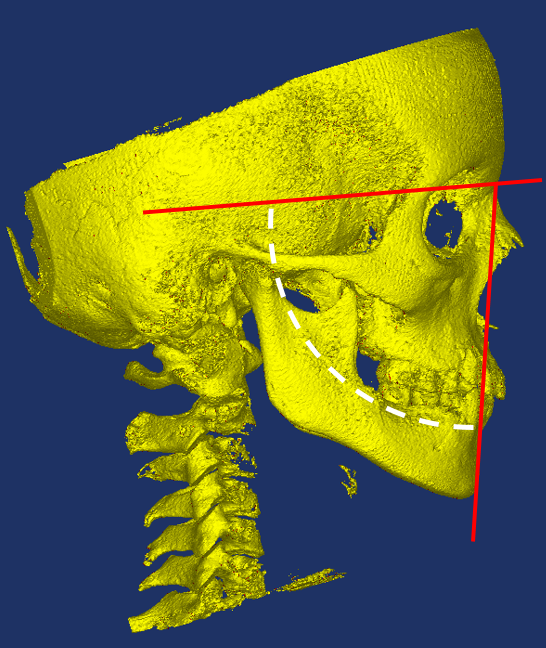


10. Mandibular occlusal inclination. The inclination of mandibular occlusal plane


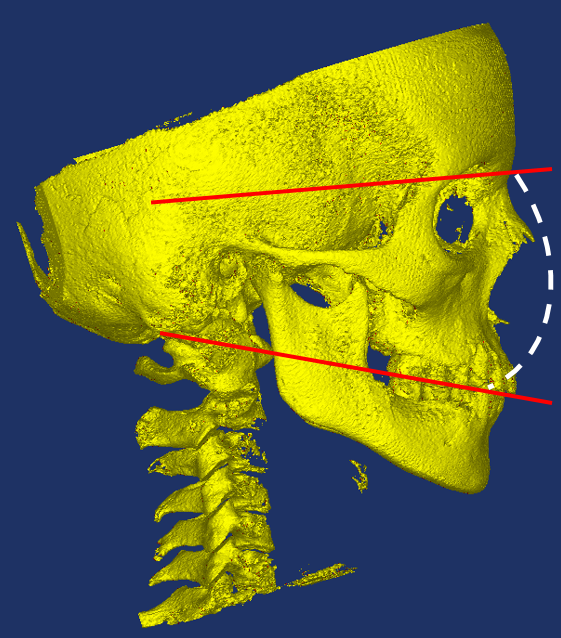


11. Anterior/posterior lower face height ratio. Anterior lower facial development


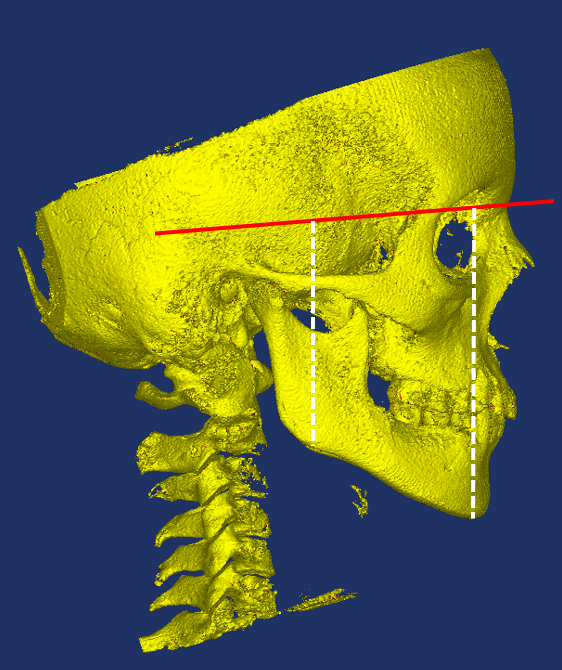


12. Wits appraisal. Difference between distance of A-point and B-point to coronal molar Construction plane


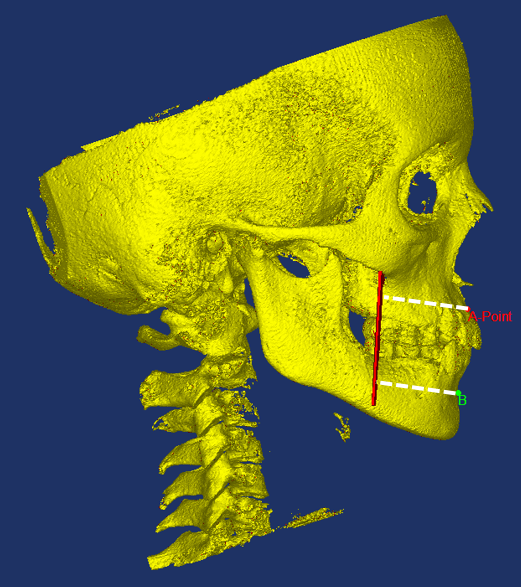


13. Transverse distance, gnathion, to midsagittal plane. Distance from gnathion to Sagittal plane


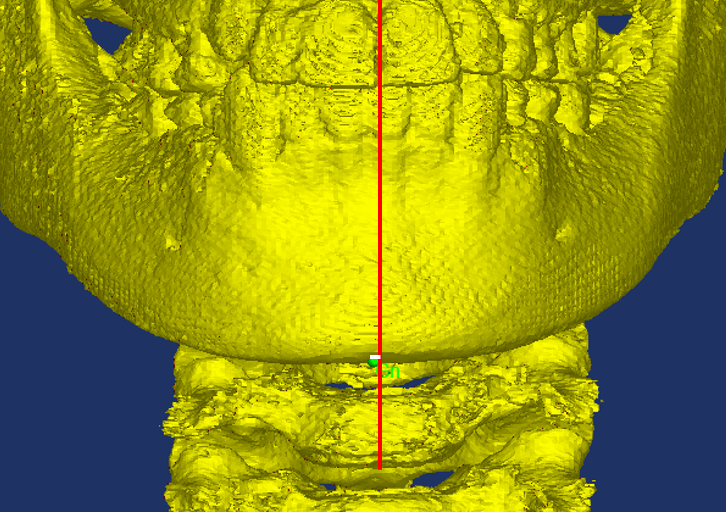


| **S4.** Reliability testing. Intra-rater values based on 30 duplicate measurements. | | | |
| --- | --- | --- | --- |
| **Reference number of morphometric measure (No.)** | **Morphometric measures** | **Intra-rater (ICC)** |  |
|  | **Inter-side diff. in bilateral linear distances** |  |  |
| 1. | Total posterior mandibular height 1 ** | 0.85 |  |
| 3. | Condylar height | 0.92 |  |
| 6. | Mandibular basal length | 0.08 |  |
| 8. | Maxillary occlusal canting ** | 0.78 |  |
| 9. | Mandibular occlusal canting ** | 0.90 |  |
|  | **Inter-side difference in bilateral angles** |  |  |
| 11. | Gonion angle | 0.81 |  |
|  | **Angles between predefined planes** |  |  |
| 12. | Mandibular axial angle ** | 0.74 |  |
| 13. | Mandibular inclination | 0.97 |  |
| 15. | Mandibular sagittal position | 0.98 |  |
| 17. | Mandibular occlusal inclination ** | 0.98 |  |
|  | **Anterior/posterior face height ratio** |  |  |
| 18. | Anterior/posterior lower face height ratio ** | 0.85 |  |
|  | **Miscellaneous** |  |  |
| 20. | Wits appraisal | 0.97 |  |
| 21. | Transverse distance, gnathion, to midsagittal plane | 0.70 |  |

#### **S5.** Inter-side differences, indicating whether the asymmetric side is consistently smaller
